# Influence of baseline magnesium concentrations on shivering in therapeutic temperature modulation

**DOI:** 10.1186/cc13692

**Published:** 2014-03-17

**Authors:** KL Johnson

**Affiliations:** 1 HCMC, Minneapolis, MN, USA

## Introduction

Therapeutic temperature modulation (TTM) is widely used in the care setting to improve outcomes of patients with traumatic brain injury (TBI). Through fever prevention, both oxygen utilization and caloric expenditure are reduced, so metabolic efficiency can be maximized [1]. However, patient cooling is not without consequences and shivering is experienced by more than 70% of patients achieving TTM. Because shivering triggers an increase in metabolic demand, causing additional oxygen consumption and the promotion of catabolism, its prevention is ideal [2]. We set out to review the data surrounding the anti-shivering component of a normothermia protocol in the surgical ICU (SICU) of one Minnesota hospital.

## Methods

A retrospective review was conducted looking at SICU patients managed with a normothermia protocol, with particular attention paid to the anti-shivering portion of the protocol. Serum magnesium (Mg) levels were assessed prior to initiation of TTM and Bedside Shivering Assessment Scale (BSAS) scores were collected.

## Results

**Figure 1:**
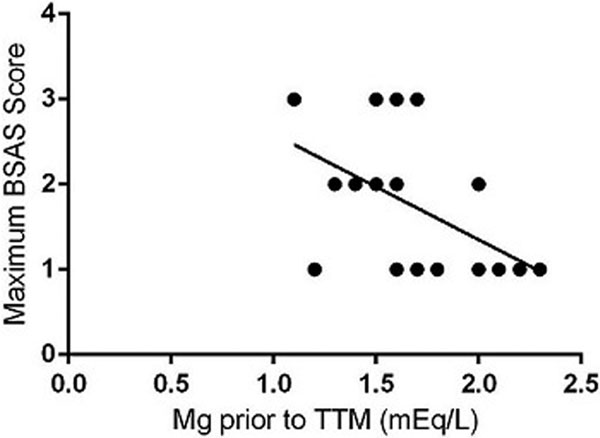


Twenty patients receiving TTM for TBI were evaluated (March to October 2013). One-half of the patients maintained targeted BSAS scores <1 for the full duration of TTM (*n *= 10 of 20). Serum Mg levels at the initiation of TTM were observed to negatively correlate with the level of shivering, as indicated by the BSAS scoring system (*P *= 0.02). See Figure 1.

## Conclusion

The literature suggests the positive impact of TTM on patient outcomes can be maximized with shivering prevention [2]. Current SICU practices provide a similar Mg loading dose for all patients, regardless of baseline Mg levels. In our observed patients, achieving a baseline serum Mg level >2 was associated with lower shivering scores throughout the TTM course. This supports the hypothesis that serum Mg concentrations prior to TTM are important predictors of shivering reduction, and suggests that loading doses of Mg should be tailored to the individual patient to achieve such levels.
